# *Schistosoma mansoni* schistosomula antigens induce Th1/Pro‐inflammatory cytokine responses

**DOI:** 10.1111/pim.12592

**Published:** 2018-10-21

**Authors:** Moses Egesa, Lawrence Lubyayi, Edridah M. Tukahebwa, Bernard S. Bagaya, Iain W. Chalmers, Shona Wilson, Cornelis H. Hokke, Karl F. Hoffmann, David W. Dunne, Maria Yazdanbakhsh, Lucja A. Labuda, Stephen Cose

**Affiliations:** ^1^ Department of Medical Microbiology School of Biomedical Sciences Makerere University College of Health Sciences Kampala Uganda; ^2^ Medical Research Council/Uganda Virus Research Institute and London School of Hygiene & Tropical Medicine Uganda Research Unit Entebbe Uganda; ^3^ Vector Control Division Ministry of Health Kampala Uganda; ^4^ Department of Immunology and Molecular Biology School of Biomedical Sciences Makerere University College of Health Sciences Kampala Uganda; ^5^ Institute of Biological, Environmental & Rural Sciences Aberystwyth University Aberystwyth UK; ^6^ Department of Pathology University of Cambridge Cambridge UK; ^7^ Department of Parasitology Leiden University Medical Center Leiden The Netherlands; ^8^ Department of Clinical Research London School of Hygiene & Tropical Medicine London UK

**Keywords:** cytokines, *Schistosoma mansoni*, schistosomula, Th1/pro‐inflammatory, vaccine

## Abstract

Larvae of *Schistosoma* (schistosomula) are highly susceptible to host immune responses and are attractive prophylactic vaccine targets, although cellular immune responses against schistosomula antigens in endemic human populations are not well characterized. We collected blood and stool from 54 *Schistosoma mansoni*‐infected Ugandans, isolated peripheral blood mononuclear cells and stimulated them for 24 hours with schistosome adult worm and soluble egg antigens (AWA and SEA), along with schistosomula recombinant proteins rSmKK7, Lymphocyte Antigen 6 isoforms (rSmLy6A and rSmLy6B), tetraspanin isoforms (rSmTSP6 and rSmTSP7). Cytokines, chemokines and growth factors were measured in the culture supernatants using a multiplex luminex assay, and infection intensity was determined before and at 1 year after praziquantel (PZQ) treatment using the Kato‐Katz method. Cellular responses were grouped and the relationship between groups of correlated cellular responses and infection intensity before and after PZQ treatment was investigated. AWA and SEA induced mainly Th2 responses. In contrast, rSmLy6B, rSmTSP6 and rSmTSP7 induced Th1/pro‐inflammatory responses. While recombinant antigens rSmKK7 and rSmLy6A did not induce a Th1/pro‐inflammatory response, they had an association with pre‐treatment infection intensity after adjusting for age and sex. Testing more schistosomula antigens using this approach could provide immune‐epidemiology identifiers necessary for prioritizing next generation schistosomiasis vaccine candidates.

## 1 INTRODUCTION

There are currently 206.4 million people suffering from schistosomiasis worldwide^1^ with the majority of infected individuals living in Africa. National control programmes in high‐risk countries such as Uganda treat infected school‐age children and adults through mass drug administration (MDA), using the drug praziquantel (PZQ). Although the MDA programmes have a high coverage in the targeted areas, national coverage still remains relatively low. For instance, in 2015 just over a third of the estimated 13.2 million people who required treatment in Uganda were reported to have received it.^1^ In addition, MDA programmes have no effect on recurrent reinfections in hotspots of transmission.^2^ More unsettlingly, there are reports of reduced efficacy of PZQ with multiple rounds of MDA.^3^, ^4^ This suggests that MDA alone is insufficient to control morbidity and prevent schistosomiasis transmission. Therefore, an integrated approach with other interventions such as vaccines is required.^5^, ^6^

A few schistosomiasis vaccines, such as *Sh*28GST, *Sm‐*TSP‐2 and *Sm*‐14 are currently in early human clinical trials,^7^, ^8^ although the trial data are not yet available. Therefore, novel schistosome vaccine antigens are still needed for the vaccine development pipeline. Schistosome larvae within the vertebrate host (known as schistosomula) are susceptible to host immune responses in animal models, and thus antigens from this stage are thought to be potential vaccine candidates.^9^, ^10^, ^11^ The schistosomula develop when infective free‐swimming fresh‐water larvae (cercariae) burrow through the host skin and lose their bifurcated tails in a process called transformation.^12^ Additional structural changes, such as the loss of the cercarial glycocalyx coat during the transformation process, expose the developing schistosomula to host‐mediated immune responses. As the skin‐stage schistosomula develop into the lung‐stage schistosomula and adult worms, they acquire host antigens^13^ masking themselves from host immune effector mechanisms.^14^ By targeting antigens from the early schistosomula, it might be possible to attack this stage and prevent infection of the host. Potential vaccine candidates expressed in the newly transformed schistosomula include SmKK7 (Smp_194830),^15^, ^16^ SmLy6A (Smp_019350) and SmLy6B (Smp_105220)^13^, ^17^ and the tetraspanins SmTSP6 (Smp_059530) and SmTSP7 (Smp_099770).^18^, ^19^

SmKK7 is secreted by both the cercariae and the schistosomula^16^; it has also been reported to be found in the nervous system of adult *S. mansoni* and to be homologous to a component in scorpion venom, acting as a potassium ion channel blocker. *Schistosoma mansoni* lymphocyte antigens, rSmLy6A and rSmLy6B (also known as SmCD59a and SmCD59b), are members of the three‐finger protein domain (TFPD) superfamily. Although they are homologous to the TFPD‐containing human CD59, which protects human cells from complement fixation, rSmLy6A and rSmLy6B do not inhibit host complement fixation and as such their function remains unknown.^20^ rSmLy6A and rSmLy6B are highly expressed by the schistosomula, and as probable GPI‐anchored proteins on the schistosome tegument, they likely interact directly with host immune cells.^21^ *Schistosoma mansoni* transmembrane proteins, tetraspanins rSmTSP6 and rSmTSP7, similarly to other tetraspanin family members, are thought to be involved in cell membrane biology.^19^, ^22^ As all of the proteins described above are at the interface between the parasite and the host immune system, and thus may be novel vaccine antigens, we assessed the cellular responses to these antigens in individuals residing in an *S. mansoni* endemic area.

## 2 MATERIALS AND METHODS

### 2.1 Ethics statement

Ethical approval for the study was obtained from the Makerere University School of Biomedical Sciences Higher Degrees Research and Ethics Committee (reference number SBS 300) and the Uganda National Council for Science and Technology (reference number HS 1040). A signed informed consent form was obtained from participants, or parents or legal guardians of children below 18 years of age, for enrolment in the study.

### 2.2 Recruitment of study participants

We examined immune responses in peripheral blood mononuclear cells (PBMCs) of participants from TheSchistoVac study (http://www.theschistovac.eu) which aimed to develop safe candidates for a prophylactic schistosomiasis vaccine. The design of TheSchistoVac study is described elsewhere.^23^ Briefly, a cohort of 372 people from an *S. mansoni* endemic fishing village, Namoni, in Mayuge District, Eastern Uganda, were recruited in September 2011; infected individuals were treated with two doses of PZQ (40 mg/kg) 1 week apart and followed up at 5 weeks and at 1 year after PZQ treatment. Venous blood was drawn before treatment and peripheral blood mononuclear cells (PBMCs) isolated by density gradient centrifugation using Lymphoprep^™^ (STEMCELL Technologies Inc, Cambridge, MA, USA) and cryopreserved in liquid nitrogen. Here, we report pre‐treatment cellular immune responses in 54 participants from the cohort described above.

### 2.3 Microscopic examination of stool for ova

Stool samples were collected before treatment (to determine pre‐treatment infection intensity), 5 weeks after treatment (to examine effectiveness of the treatment) and at 1 year after PZQ treatment (to determine reinfection). Three stool samples were collected on three consecutive days from study participants. Two thick smears were made from each of the stool samples, processed using the Kato‐Katz method^24^ and examined under a light microscope to determine *S. mansoni* egg count.

### 2.4 *Schistosoma mansoni* antigens

TheSchistoVac consortium provided the antigens used in this study. The antigens included *S. mansoni* adult worm antigen (AWA)^25^ and *S. mansoni* soluble egg antigen (SEA)^25^ as well as the *S. mansoni* schistosomula‐enriched recombinant antigens rSmKK7 (smp_194830), rSmLy6A (smp_019350), rSmLy6B (smp_105220), rSmTSP6 (smp_059530) and rSmTSP7 (smp_099770).^19^ The antigens used in the present study are summarized in Table 1. These antigens were identified as highly expressed products in the schistosomula life cycle stage after screening the *Schistosoma* transcriptome using a DNA microarray, as previously described.^17^, ^19^ Specifically, a >5‐fold increase in expression when comparing normalized expression averages from snail (egg, miracidia, mother sporocyst and daughter sporocyst) to schistosomula (3‐, 24‐hours, 3‐ and 6‐day schistosomula) life stages was observed.^17^ Recombinant antigens were expressed in *Escherichia coli* and purified using methods as previously described for rSmKK7,^14^ rSmLy6A and rSmLy6B.^15^ For rSmTSP6 and rSmTSP7, 84 and 78 amino acids were expressed, representing the extracellular loop 2 of these proteins, respectively, as defined by TMHMM2.0 software.^26^ About 106‐189 amino acid of rSmTSP6 and 108‐185 amino acid of rSmTSP7 were expressed using the same vector, expression parameters and purification methods as rSmLy6A and B.^27^ Endotoxin contamination of the recombinant antigens was determined by Limulus amebocyte lysate (LAL) assay using the Pierce LAL chromogenic endotoxin quantitation kit (Thermo Fisher, Pittsburgh, PA, USA). The endotoxin levels were 7.56 endotoxin units (EU)/mg for rSmKK7, undetectable (<0.4 EU/mg) for rSmLy6A, 0.892 EU/mg for rSmLy6B, 0.626 EU/mg for rSmTSP6 and 0.42 EU/mg for rSmTSP7. The limit of detection of the LAL assay was 0.4 EU/mg.

**Table 1:** The list of recombinant antigens used in the present study

Name	Alternative names	*Schistosoma mansoni* GeneDB identification^a^	Predictions from protein data	Protein size (amino acids)^d^	References
SmKK7		Smp_194830	Signal peptide predicted^b^ No transmembrane regions predicted^c^	79	15, 16
SmLy6a	SmCD59a, SmCD59.1	Smp_019350	One transmembrane regions predicted^c^	126	13, 20
SmLy6b	SmCD59b, SmCD59.2	Smp_105220	One transmembrane regions predicted^c^	124	13, 20, 21
SmTSP6		Smp_059530	Four transmembrane regions predicted^c^	196	18, 19
SmTSP7		Smp_099770	Four transmembrane regions predicted^c^	225	18, 19

a GeneDB is an annotation database for pathogens.^58^

b Protein data retrieved from GeneDB.

c Determined using SignalP.

d Determined using TMHMM.

### 2.5 PBMC stimulation

Peripheral blood mononuclear cells were thawed, rested for 6 hours and stimulated for 24 hours with a panel of *S. mansoni* antigens in RPMI 1640 Medium (Thermo Fischer): AWA (10 μg/mL), SEA (10 μg/mL), rSmKK7 (2 μg/mL), rSmLy6A (2 μg/mL), rSmLy6B (2 μg/mL), rSmTSP6 (2 μg/mL) and rSmTSP7 (2 μg/mL). Due to a limited number of PBMCs available per individuals and a large number of antigens tested, each antigen was tested in singlicate. After 24 hours, the supernatants were collected and stored at −80°C. Medium without stimulus was used as a negative control.

### 2.6 Multiplex luminex assay

Supernatants were analysed for cytokines (IFN‐γ, L‐1β, IL‐1ra, IL‐2, IL‐4, IL‐5, IL‐6, IL‐7, IL‐9, IL‐10, IL‐12, IL‐13, IL‐15, IL‐17 and TNF), chemokines (Eotaxin, IL‐8, IP‐10, MCP‐1, MIP1a, MIP1b, RANTES) and growth factors (bFGF, GCSF, GMCSF, PDGFbb, VEGF) using a commercial Bio‐Plex Luminex assay (Bio‐Rad, Hercules, CA, USA) according to the manufacturers’ recommendations, and acquired using Bio‐Plex 200 System (Bio‐Rad). Samples below the limit of detection were assigned values corresponding to half of the lowest standard value and those above the highest limit of detection were given the value of the highest standard. Cytokines that were wholly below the lower limit of the assay (IL‐4 and IL‐7) and chemokines wholly above the upper limit of the assay (MIP1α, MIP1β and RANTES) were excluded from analysis. Samples from unstimulated PBMCs that produced TNF levels >100 pg/mL were also excluded from analysis, as these were considered unreliable^28^, ^29^; high IL‐1β and IL‐1ra levels were observed in the same samples.

### 2.7 Statistical data analysis

Statistical analysis was carried out using STATA version 13 (StataCorp, College Station, TX, USA) and graphs drawn using GraphPad Prism version 6.0g (GraphPad Software Inc., San Diego, CA, USA). Cytokine, chemokine and growth factor levels were not normally distributed and were, therefore, transformed using Box‐Cox transformation.^30^, ^31^ The paired Student's *t* test was used to compare responses between antigen stimulated and unstimulated PBMCs. Because the mean cytokine/chemokine/growth factor levels of more than two antigen‐stimulated PBMCs was compared to that of the unstimulated PBMCs, the error rate due to multiple testing was adjusted by considering a Bonferroni correction, taking into account the number of comparisons being made. In this instance, a *P*‐value of 0.007 (= 0.05/7, the number of antigens in the study) for each cytokine/chemokine/growth factor was taken to be statistically significant. Responses showing significant variation from the unstimulated PBMCs were further analysed. Unsupervised hierarchical clustering of the immune responses using Spearman correlation as the measure of similarity was performed using GENE‐E (Broad Institute Inc., Cambridge, MA, USA). The association between clusters (groups of correlated immune parameters) and infection intensity before treatment and following treatment was investigated using the global test version 5.29.1. The global test was based on the logistic regression model and was implemented in R version 3.3.2 (The R Foundation, Vienna, Austria). For this analysis, the post‐treatment infection intensity of the participants was classified into two groups: light, and moderate to heavy infection intensity, rather than the WHO categories (light/moderate/heavy infection intensity), as when stratified by the three WHO categories there were insufficient numbers within the moderate and heavy infection intensity groups for statistical analysis.

## 3 RESULTS

### 3.1 Characteristics of the study population

Table 2 shows the demographics of the study population. The age range of the study participants was 6‐40 years and 28 (51.9%) were female. All study participants were infected with *S. mansoni* with most individuals having heavy infection intensity (76%) at baseline. The median infection intensity was 1412 (interquartile range 443‐2567) eggs per gram of stool at baseline. Five weeks after PZQ treatment, all the participants had zero eggs, indicative of treatment success (data not shown). At follow‐up (1 year after PZQ treatment), all participants were re‐infected with most (57.0%) having a light infection intensity.

**Table 2:** Characteristics of study population (N = 54)

Age, median (range)	12 (6‐40)
Females, n (%)	28 (52)
Pre‐treatment infection intensity	
Light (1‐99 epg), n (%)	7 (13)
Moderate (100‐399 epg), n (%)	6 (11)
Heavy (400+ epg), n (%)	41 (76)
1‐year follow‐up infection intensity	
Light (1‐99 epg), n (%)	31 (57)
Moderate to heavy (100+ epg), n (%)	23 (43)

epg, eggs per gram.

### 3.2 *Schistosoma mansoni* adult worm and egg antigens induce Th2 responses

**Figure 1:**
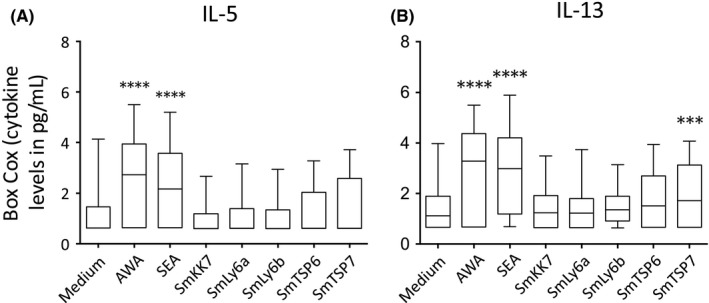
Box‐Cox transformed Th2 cytokine levels in response to stimulation of PBMCs from *Schistosoma mansoni*‐infected participants (n = 54) before PZQ treatment with AWA, SEA and schistosomula antigens compared with medium (A) IL‐5 (B) IL‐13. Box and whisker plots show median, interquartile range, maximum and minimum of cytokine levels. A paired Student's *t* test was used to test differences between medium and antigens. *P < 0.05, **P < 0.007, ***P < 0.001, ****P < 0.0001

Whole parasite preparations of *S. mansoni,* particularly the egg antigens, are known to induce Th2 responses.^27^, ^28^, ^29^ We sought to validate our protocol by examining immune responses to crude parasite preparations of AWA and SEA. As expected, and confirming our analysis approach, the adult worm and soluble egg antigens induced the production of significant levels of IL‐5 and IL‐13 compared to medium (Figure 1). None of the schistosomula antigens induced significant Th2 cytokine production, with the exception of rSmTSP7 induced IL‐13.

### 3.3 Recombinant rSmLy6B, rSmTSP6 and rSmTSP7 induce a Th1/Pro‐inflammatory cytokine profile

**Figure 2:**
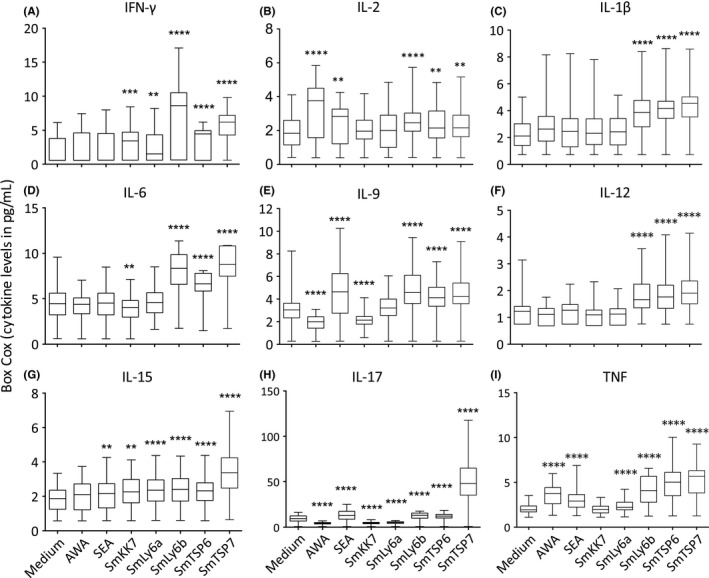
Box‐Cox transformed Th1 (A, B) and pro‐inflammatory (C‐I) cytokine levels in response to stimulation of PBMCs from *Schistosoma mansoni*‐infected participants (n = 54) before PZQ treatment with AWA, SEA and schistosomula antigens compared with medium. Box and whisker plots show median, interquartile range, maximum and minimum of cytokine levels. A paired Student's *t* test was used to test differences between medium and antigens. *P < 0.05, **P < 0.007, ***P < 0.001, ****P < 0001

**Figure 3:**
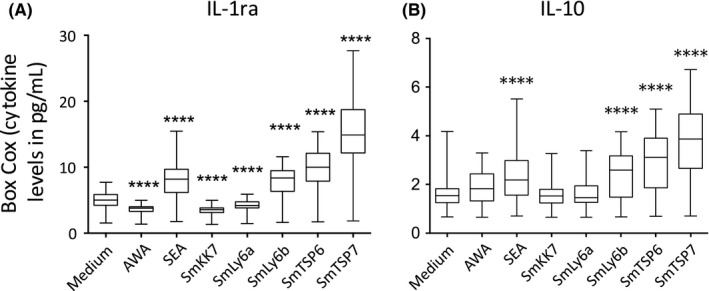
Box‐Cox transformed regulatory cytokine levels in response to stimulation of PBMCs from *Schistosoma mansoni*‐infected participants (n = 54) before PZQ treatment with AWA, SEA and schistosomula antigens compared with medium (A) IL‐1ra and (B) IL‐10. Box and whisker plots show median, interquartile range, maximum and minimum of cytokine levels. A paired Student's *t* test was used to test differences between medium and antigens. *P < 0.05, **P < 0.007, ***P < 0.001, ****P < 0.0001

Recombinant schistosomula antigens rSmLy6B, rSmTSP6 and rSmTSP7 induced significant and robust pre‐treatment cytokine responses characterized by Th1 (IFN‐γ), pro‐inflammatory (IL‐1β, IL‐2, IL‐6, IL‐9, IL‐12, IL‐15, IL‐17 and TNF) (Figure 2) and regulatory responses (IL‐1ra and IL‐10) compared to medium (Figure 3). Moreover, rSmLy6B, rSmTSP6 and rSmTSP7 induced high levels of chemokines (Figure S1) and growth factors (Figure S2). Interestingly, rSmKK7 and rSmLy6A in contrast were associated with much lower responses and suppressed production of IL‐17, IL‐1ra, bFGF, GMCSF, GCSF compared to medium (Figures 2 and 3, Figures S1 and S2).

### 3.4 Cytokine, chemokine and growth factor responses to schistosomula antigens cluster depending on the antigen

To provide a more global assessment of responses to schistosomula antigens in *S. mansoni*‐infected individuals, cytokine responses were grouped together using Spearman correlation as a similarity measure, to reduce the number of variables for statistical analyses.

**Figure 4:**
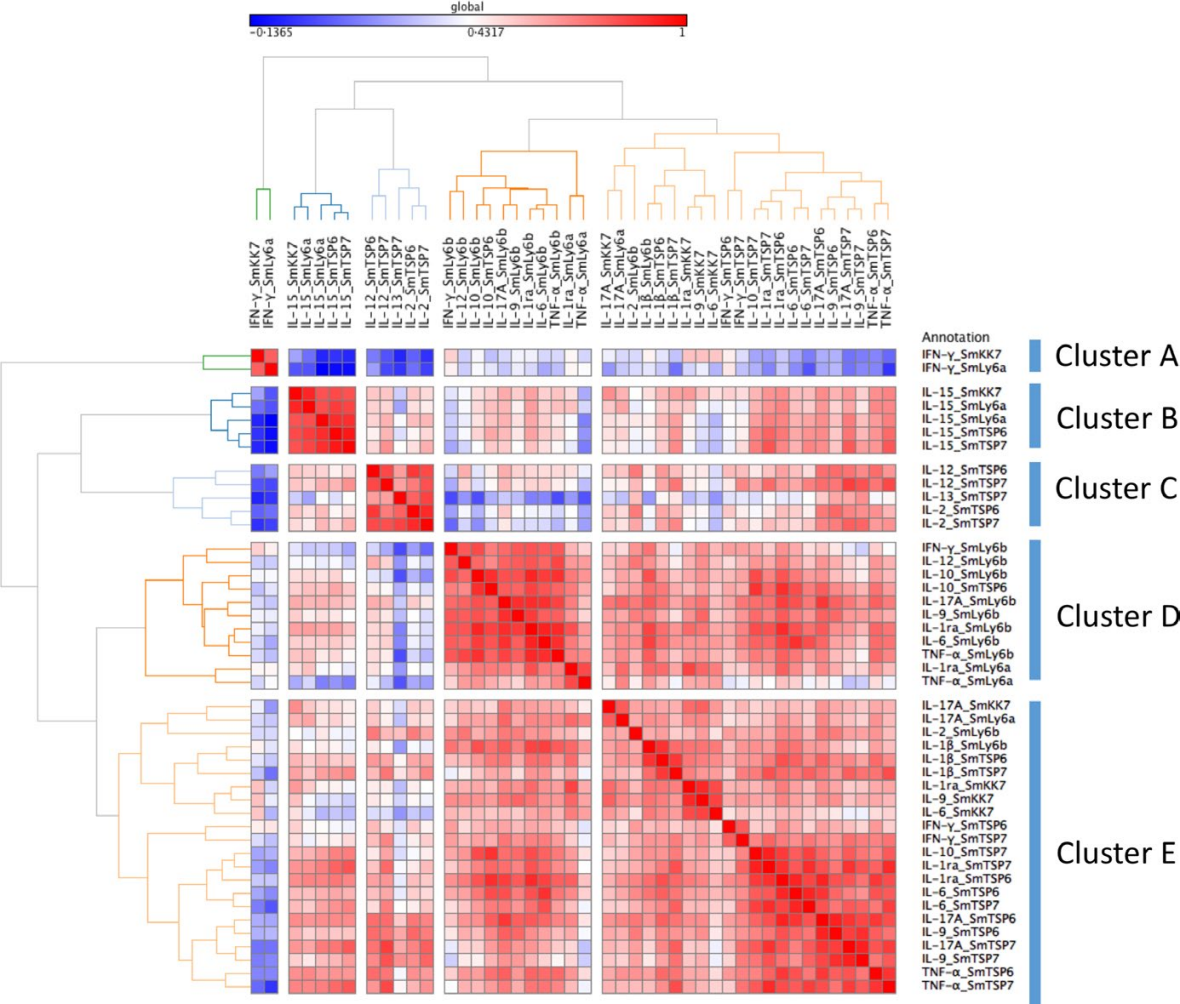
Unsupervised hierarchical clustering of cytokine responses from PBMCs of *Schistosoma mansoni*‐infected participants (n = 54) before PZQ treatment stimulated with *S. mansoni* schistosomula antigens. Red and blue colours indicate strong positive and negative correlations respectively

Cytokine responses directed against schistosomula recombinant proteins grouped into six clusters: two clusters composed of individual cytokines (an IFN‐γ only cluster A and IL‐15 only cluster B); two clusters composed of Th1/pro‐inflammatory/regulatory response responses to rSmTSP6 and rSmTSP7 (clusters C and F); a cluster composed mainly of Th1/pro‐inflammatory/regulatory response mainly to rSmLy6B (cluster D); and a pro‐inflammatory cluster in response to rSmKK7, SmLy6A and SmLy6B (cluster E) (Figure 4).

Similarly, chemokine responses to the schistosomula antigens clustered around individual chemokines (clusters A and C) and an eotaxin/IL‐8 cluster in response to all antigens tested (Figure S3A).

Finally, growth factor responses clustered around responses to rSmKK7 and rSmLy6A (cluster A), responses to rSmLy6B, rSmTSP6 and rSmTSP7 (cluster B) and a single growth factor cluster for PDGFFbb (cluster C) (Figure S3B).

### 3.5 Responses to rSmKK7 and rSmLy6A are positively associated with pre‐treatment infection intensity

To explore whether clusters of pre‐treatment cellular responses were associated with infection intensity before or 1 year post‐treatment, the global test was performed.^32^ This test examines the association between clusters (eg, groups of correlated immune parameters) and an outcome (eg, pre‐treatment or post‐treatment infection intensity). After adjusting for age and sex, a significant positive association between pre‐treatment infection intensity and responses to rSmKK7 and rSmLy6a was found for the IFN‐γ cytokine cluster (Table 3, *P* = 0.026), for the eotaxin chemokine cluster (Table S1, *P* = 0.020)) and a combination of growth factors (Table S2, *P* = 0.015). No significant associations were found between clusters of immune response and 1‐year post‐treatment infection intensity (Table 3, Tables S1 and S2).

**Table 3:** Association between clusters of cytokine responses to schistosomula antigens and pre‐treatment infection intensity

Cluster^a^	Pre‐treatment infection intensity^b^		1‐Year post‐treatment infection intensity^b^	
	Crude	Adjusted^c^	Crude	Adjusted^c^
A	**0.010** d	**0.026** d	0.060	0.131
B	0.663	0.746	0.550	0.853
C	0.189	0.312	0.178	0.363
D	0.496	0.134	0.262	0.481
E	0.307	0.140	0.810	0.946
F	0.578	0.427	0.912	0.866

a Clusters are shown in Figure 4.

b Global test *P*‐value.

c Adjusted for age and sex.

d Positive direction of association.

Bold text represents a significant difference.

## 4 DISCUSSION

The schistosomula are vulnerable to host immune responses after transformation from the cercariae stage.^33^, ^34^ As a result, antigens of the early schistosomula have been suggested as potential vaccine candidates.^21^, ^35^ Here we measured cytokine, chemokine and growth factor responses to a selection of *S. mansoni* schistosomula‐enriched antigens in endemic subjects with high infection intensity, and who become re‐infected 1 year after successful treatment.

To validate our study, we first looked at immune responses to the crude parasite antigen preparations AWA and SEA. In line with previous studies amongst individuals from high endemicity areas,^36^, ^37^, ^38^ we found predominant Th2 cytokine responses characterized by high IL‐5 and IL‐13.

Few schistosomula antigens have been tested in *S. mansoni* endemic populations. A non‐inclusive list of these antigens includes Sm29 (also known as SmLy6D)^39^ and Sm14^40^, ^41^ that have both been tested in Brazil. We found that three of the recombinant schistosomula antigens tested (rSmLy6B, rSmTSP6 and rSmTSP7) induced strong Th1/pro‐inflammatory and regulatory responses while two others (rSmKK7, and rSmLy6A) induced low responses in a Ugandan *S. mansoni* endemic population. Th1 and pro‐inflammatory cytokines have been implicated in antibody‐independent killing of schistosomula by stimulating macrophages to produce nitric oxide that mediates the killing of schistosomula in animal models.^42^, ^43^ Although the observed cytokine responses are consistent with a previous study in which a recombinant schistosomulum antigen, rSm14, induced significant levels of Th1/pro‐inflammatory cytokines at 72 hours after stimulation,^40^ our study shows that these responses can be measured earlier at 24 hours. An early response to the schistosomulum is necessary due to its transient nature in the tissues including the skin.

The generation of Th1 responses is associated with protective immunity to *S. mansoni*.^44^ For instance, the schistosomula antigen SmLy6D (Sm29) induces Th1 and pro‐inflammatory cytokines (IFN‐γ, TNF and IL‐12) that protect vaccinated mice against challenge infection.^45^ Furthermore, vaccinating mice with a chimera of two recombinant schistosomula antigens SmTSP2 and SmLy6D (Sm29) formulated in CpG‐Alum also protects vaccinated mice against infection.^46^ Whether the schistosomula antigens used in the present study, SmLy6B, SmTSP6 and SmTSP7, are protective in animal models of schistosomiasis remains to be determined. In a study in Brazil, Sm14‐specific Th1 responses were produced by PBMCs of people who were resistant to reinfection with schistosomiasis.^40^ This suggests that high antigen‐specific Th1 responses may play a protective role in human resistance to reinfection. However, with no clear resistant group in our cohort 1‐year post‐treatment, we instead looked at the association between cytokine responses and infection intensity before and after treatment with PZQ. We found no association between infection intensity at either pre‐ or post‐treatment and that Th1/pro‐inflammatory cytokines to rSmLy6B, rSmTSP6 and rSmTSP7 suggesting that in our cohort these responses may not be protective in humans and other schistosomula antigenic targets should be explored.

Recombinant SmLy6B, rSmTSP6 and rSmTSP7 induced production of regulatory cytokines IL‐10 and IL‐1ra. This is consistent with work done with another schistosomula tegument antigen, SmLy6D (Sm29), that induced IL‐10 production in PBMCs of *S. mansoni*‐infected individuals.^41^ In fact, the expression of SmLy6D (Sm29) is similar to that of rSmLy6B^16^, ^19^ and rSmTSP6 and rSmTSP7.^19^ The concomitant regulatory responses induced by the recombinant antigens in the present study may be needed to balance effector responses to limit immunopathology. This suggests that antigens rSmLy6B, rSmTSP6 and rSmTSP7 that induce regulated Th1 responses might be good targets to consider further as prospective human vaccine candidates in combination with adjuvants to induce a stronger Th1 response. In the present study, rSmKK7 did not significantly produce IL‐10. However, IL‐10 has been shown to be elevated in patients stimulated with cercarial excreted‐secreted (ES) products^47^ of which SmKK7 is a component.^16^ This inconsistency suggests that other constituents of cercarial ES material could be the major stimulants of IL‐10 production. In fact, glycosylated components of cercarial ES may play a role in the production of IL‐10.^47^ In addition, the anomaly in IL‐10 production could be caused by a difference in the stimulant used. Turner and colleagues used native cercarial ES material released from transforming cercariae,^47^ while the SmKK7 used in the present study is a purified recombinant protein without glycosylated moieties.

Interestingly, rSmTSP7 induced IL‐13 production in stimulated PBMCs of infected participants, whereas the other schistosomula antigens did not. In addition, the same rSmTSP7 induced Th1/pro‐inflammatory cytokine production. These findings imply a mixed Th1/Th2 response to recombinant schistosomula antigen SmTSP7. As much as murine studies have suggested that Th1 responses against migrating larvae may be more host protective than Th2 responses,^46^, ^48^, ^49^ a mixed Th1/Th2 response in mice vaccinated with radiation‐attenuated cercariae indicates that induction and balance of both Th1 and Th2 are required for vaccine‐induced protection and not a highly polarized Th1 or Th2.^50^ Unregulated and highly polarized immune responses are responsible for the immunopathology associated with schistosomiasis in mice^51^ and humans.^52^ This property of rSmTSP7 to induce a regulated mixed Th1/Th2 response positions it as a potential vaccine candidate to investigate further.

Recombinant rSmKK7 and rSmLy6A induced low responses and suppressed the production of IL‐17 and IL‐1ra compared to PBMCs cultured in medium alone. rSmKK7 shares homology with a venom protein BmKK7 of the Asian scorpion *Buthus martensi* Karsch (BmK) that blocks potassium channels.^16^, ^53^ As potassium channels are vital in the regulation of T cell activation after antigenic stimulation,^54^ it is possible that SmKK7 affects potassium channels, thereby modulating T cell activation and suppressing cytokine production by T cells.^16^ It remains to be experimentally determined if indeed SmKK7 modulates human T cell activation. Furthermore, Th1/pro‐inflammatory immune responses to rSmKK7 and rSmLy6A were positively associated with pre‐treatment infection intensity. However, the suitability of these antigens as vaccine candidates is debatable because they elicited low responses and were not associated with protection against *S. mansoni* reinfection.

The cytokine responses to the recombinant antigens observed in this study likely reflect the differences in the expression of the gene products of the schistosomula antigens, and their localisation within the *S. mansoni* organism. SmLy6B, SmTSP6 and SmTSP7 are localized in the tegument and their gene products are highly expressed during the 3‐, 24‐hour, 3‐ and 6‐day schistosomula as well as adult life stages^17^, ^19^; SmLy6A, also a tegument antigen, is only highly expressed in the 6‐day schistosomula, while SmKK7 is a secreted molecule.^16^

Because of the small number of participants in the present analysis, a larger study may be warranted to investigate the association between cytokine responses to the schistosomula antigens and infection intensity 1 year after PZQ treatment. Nonetheless, a large number of cytokines were evaluated per participant. While this creates a problem of multiple comparisons, the approach taken in the present study using unsupervised hierarchical clustering allows for large numbers of cytokines/ growth factors/chemokines to be tested as a group rather than as individual parameters. The responses to the crude antigens AWA and SEA were not represented in the cluster analysis of response to recombinant schistosomula antigens. The global test in the present study utilised the groups of cytokines/growth factors/chemokines to find a relationship with either pre‐treatment or 1‐year post‐treatment infection intensity. The global test allowed for a global analysis of responses which may give broader and generalized insights. The global test has been used elsewhere to study clusters of microarray gene data and how the gene clusters relate to two types of leukaemia.^32^ In addition, the global test adjusts for multiple comparisons since few groups are tested.^32^ In essence, the global test may be useful for studies of cohorts with many independent measurements.

The rate of exposure to the *Schistosoma* through water contact is associated with resistance to reinfection with schistosomiasis.^55^, ^56^, ^57^ It follows that exposure history during follow‐up is an important variable in the understanding of reinfection with schistosomiasis. The participants in the present study might have had varied rates of exposure during follow‐up, which was not assessed in the present study and thus affecting immune responses to schistosomula antigens. To control exposure to infection in future, immunogenicity studies of vaccine antigens, a controlled human infection model (CHIM) for schistosomiasis, could be explored. Hitherto the hookworm was the only human helminth to be studied in CHIM for its potential as a vaccine (NCT01940757). The first CHIM for schistosomiasis is ongoing with European volunteers using a single sex (male cercariae) infection to assess safety and optimal dose of such controlled infections (NCT02755324). Based on the findings from this trial, future CHIM for schistosomiasis could investigate human responses to promising *Schistosoma* antigens including schistosomula antigens in endemic populations.

In conclusion, our findings show that the schistosomula antigens rSmLy6B, rSmTSP6 and rSmTSP7 induce a predominantly Th1/pro‐inflammatory response. The recombinant antigens SmLy6B, SmTSP6 and SmTSP7 might be good targets to investigate further as vaccine antigens against *S. mansoni* infection.

## CONFLICT OF INTEREST

The authors declare that there is no conflict of interest.
