# Supplementary material for: Schistosoma mansoni schistosomula antigens induce Th1/Pro‐inflammatory cytokine responses
Source: Parasite Immunol. 2018 Oct 21;40(12):e12592. doi: 10.1111/pim.12592 (PMC6492251; doi:10.1111/pim.12592)
Supplement: Supplementary file 1 [file PIM-40-na-s001.doc]

**Figure S1** Box-Cox transformed chemokine levels in responses to stimulation of PBMCs from *S. mansoni* infected participants (n=54) before PZQ treatment with AWA, SEA and schistosomula antigens compared with medium (A) IP-10 (B) IL-8 (C) Eotaxin (D) MCP-1. Box and whisker plots show median, interquartile range, maximum and minimum of cytokine levels. A paired Student’s T-test was used to test differences between medium and antigens. * p<0.05, ** p<0.007, *** p<0.001, **** p<0.0001
