# Supplementary material for: Schistosoma mansoni schistosomula antigens induce Th1/Pro‐inflammatory cytokine responses
Source: Parasite Immunol. 2018 Oct 21;40(12):e12592. doi: 10.1111/pim.12592 (PMC6492251; doi:10.1111/pim.12592)
Supplement: Supplementary file 2 [file PIM-40-na-s002.doc]

**Figure S2** Box-Cox transformed growth factor levels in response to stimulation of PBMCs from *S. mansoni* infected participants (n=54) before PZQ treatment with AWA, SEA and schistosomula antigens compared with medium (A) bFGF (B) GMCSF (C) GCSF (D) PDGFbb (E) VEGF. Box and whisker plots show median, interquartile range, maximum and minimum of cytokine levels. A paired Student’s T-test was used to test differences between medium and antigens. * p<0.05, ** p<0.007, *** p<0.001, **** p<0.0001
