# Supplementary material for: Schistosoma mansoni schistosomula antigens induce Th1/Pro‐inflammatory cytokine responses
Source: Parasite Immunol. 2018 Oct 21;40(12):e12592. doi: 10.1111/pim.12592 (PMC6492251; doi:10.1111/pim.12592)
Supplement: Supplementary file 3 [file PIM-40-na-s003.doc]

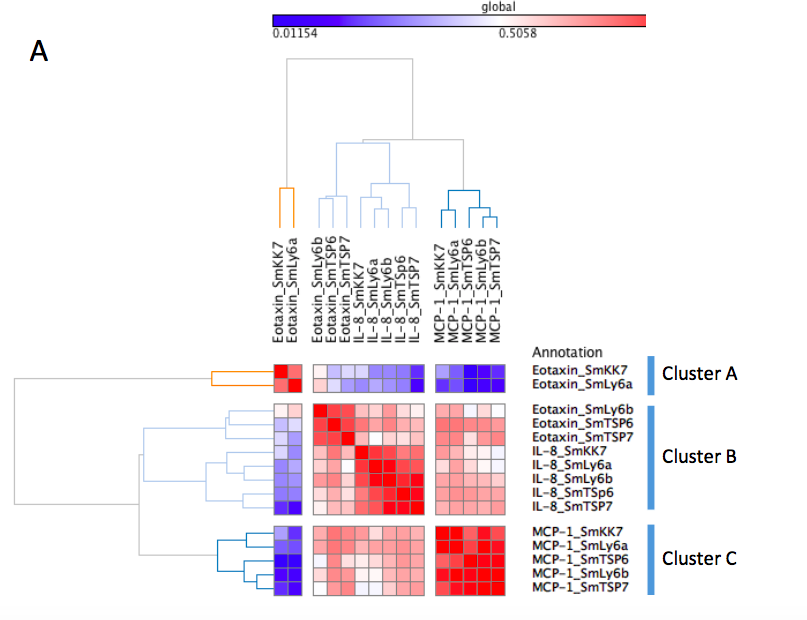


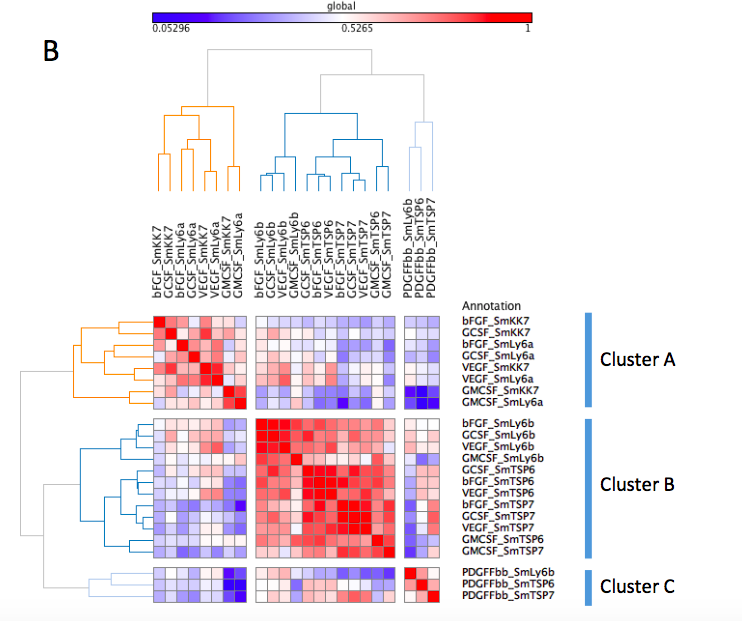


**Figure S3** Unsupervised hierarchical clustering of (A) chemokine and (B) growth factor responses from PBMCs of *S. mansoni* infected participants (n=54) before PZQ treatment stimulated with to *S. mansoni* schistosomula antigens using Spearman correlation as a measure of similarity. Red and blue colors indicate strong positive and negative correlations respectively
