# Supplementary material for: Schistosoma mansoni schistosomula antigens induce Th1/Pro‐inflammatory cytokine responses
Source: Parasite Immunol. 2018 Oct 21;40(12):e12592. doi: 10.1111/pim.12592 (PMC6492251; doi:10.1111/pim.12592)
Supplement: Supplementary file 4 [file PIM-40-na-s004.docx]

**Supplementary Tables**

Table S1 Association Between Clusters of Chemokine Responses to Schistosomula Antigens and Infection Intensity

| Cluster† | Pre-treatment Infection Intensity‡ | | 1-Year Post-treatment  Infection Intensity‡ | |
| --- | --- | --- | --- | --- |
|  | Crude | Adjusted^§^ |  | Crude |
| A | 0.054^¶^ | **0.020**^¶^ | 0.809 | 0.890 |
| B | 0.812 | 0.351 | 0.434 | 0.656 |
| C | 0.821 | 0.830 | 0.718 | 0.811 |

† Clusters are shown in Figure S3A

‡ Global Test p-value

§ Adjusted for age and sex

¶ Positive direction of association

Table S2 Association Between Clusters of Growth Factor Responses to Schistosomula Antigens and Infection Intensity

| Cluster† | Pre-treatment  Infection Intensity‡ | | 1-Year Post-treatment  Infection Intensity‡ | |
| --- | --- | --- | --- | --- |
|  | Crude | Adjusted^§^ |  | Crude |
| A | **0.012**^¶^ | **0.015**^¶^ | 0.777 | 0.935 |
| B | 0.407 | 0.160 | 0.526 | 0.757 |
| C | 0.718 | 0.626 | 0.560 | 0.805 |

† Clusters are shown in Figure S3B

‡ Global Test p-value

§ Adjusted for age and sex

¶ Positive direction of association
